# Estimating carbon fixation of plant organs for afforestation monitoring using a process‐based ecosystem model and ecophysiological parameter optimization

**DOI:** 10.1002/ece3.5328

**Published:** 2019-06-26

**Authors:** Tatsuya Miyauchi, Takashi Machimura, Makoto Saito

**Affiliations:** ^1^ Center for Global Environmental Research National Institute for Environmental Studies Tsukuba Japan; ^2^ Division of Sustainable Energy and Environmental Engineering, Graduate School of Engineering Osaka University Osaka Japan

**Keywords:** afforestation, carbon cycle, Clean Development Mechanism, monitoring, parameter optimization, process‐based ecosystem model

## Abstract

Afforestation projects for mitigating CO_2_ emissions require to monitor the carbon fixation and plant growth as key indicators. We proposed a monitoring method for predicting carbon fixation in afforestation projects, combining a process‐based ecosystem model and field data and addressed the uncertainty of predicted carbon fixation and ecophysiological characteristics with plant growth. Carbon pools were simulated using the Biome‐BGC model tuned by parameter optimization using measured carbon density of biomass pools on an 11‐year‐old *Eucommia ulmoides* plantation on Loess Plateau, China. The allocation parameters fine root carbon to leaf carbon (FRC:LC) and stem carbon to leaf carbon (SC:LC), along with specific leaf area (SLA) and maximum stomatal conductance (*g*_smax_) strongly affected aboveground woody (AC) and leaf carbon (LC) density in sensitivity analysis and were selected as adjusting parameters. We assessed the uncertainty of carbon fixation and plant growth predictions by modeling three growth phases with corresponding parameters: (i) before afforestation using default parameters, (ii) early monitoring using parameters optimized with data from years 1 to 5, and (iii) updated monitoring at year 11 using parameters optimized with 11‐year data. The predicted carbon fixation and optimized parameters differed in the three phases. Overall, 30‐year average carbon fixation rate in plantation (AC, LC, belowground woody parts and soil pools) was ranged 0.14–0.35 kg‐C m^−2^ y^−1^ in simulations using parameters of phases (i)–(iii). Updating parameters by periodic field surveys reduced the uncertainty and revealed changes in ecophysiological characteristics with plant growth. This monitoring method should support management of afforestation projects by carbon fixation estimation adapting to observation gap, noncommon species and variable growing conditions such as climate change, land use change.

## 1 INTRODUCTION

Planted forests, including both forest plantations and rubber plantations but not oil‐palm plantations or other agricultural plantations, account for 7% of global forest area (FAO, 2015). Although natural forest area declined from 3,961 Mha in 1990 to 3,721 Mha in 2015, planted forest area increased from 168 Mha to 278 Mha over the same period, helping to mitigate the annual rate of net forest loss (Keenan et al., 2015). Afforestation and reforestation could reduce the harvest pressure on natural forests by supplying timber (Fenning & Gershenzon, 2002) and reduce surface runoff and soil erosion in degraded landscapes through rainfall interception (Nunes, Almeida, & Coelho, 2011). Forest management using afforestation and reforestation is also an effective tool for promoting carbon dioxide (CO_2_) absorption through plant regrowth (Keith, Mackey, & Lindenmayer, 2009; Noormets et al., 2015; Xu, Wen, Zhu, & He, 2017). Indeed, the recent promotion of afforestation and reforestation provides carbon storage, resulting in an increase of net CO_2_ uptake by the forests in China (Streets et al., 2001). Afforestation and reforestation therefore are mitigation and adaptation strategies for climate change in the forest sector (Ravindranath, 2007).

Afforestation projects under market mechanisms in the Kyoto Protocol and the Paris Agreement aim to reduce CO_2_ while supporting sustainable development. The Clean Development Mechanism (CDM) is part of the emerging carbon market established under the terms of the Kyoto Protocol and aims to achieve sustainable development in developing countries and cost‐effective reduction of greenhouse gases in developed countries (Olsen, 2007). Olsen (2007) classified CDM projects into a typology of four groups according to their key findings: forward‐looking studies, sustainability impact studies, carbon forestry studies, and mixed studies. Afforestation and reforestation are accepted as eligible activities in the carbon forestry studies of CDM projects. Globally, more than 760 Mha of land are identified as biophysically suitable for CDM afforestation and reforestation (CDM‐AR) activities (Zomer, Trabucco, Bossio, & Verchot, 2008). In mid‐2009, however, only four projects were registered as CDM‐AR with the United Nations Framework Convention on Climate Change (UNFCCC) out of a total of 1,665 registered projects (Thomas, Dargusch, Harrison, & Herbohn, 2010). The bottlenecks in the development of CDM‐AR projects can be attributed to both the length of time it takes to gain revenue from the project and a lack of the knowledge and technical capacity required to meet the demands of the CDM registration process (Thomas et al., 2010).

In the approved methodology for CDM‐AR, it is stipulated that projects will monitor changes in carbon stocks in five pools after afforestation or reforestation—aboveground biomass, belowground biomass, dead wood, litter, and soil organic carbon—based on quality assurance/quality control (QA/QC) procedures for inventory operations (UNFCCC, 2013). The more advanced methods in the guide recommend the application of direct measurements of the carbon stock growth rate and its validation using modeling approaches (Penman et al., 2000), allowing an application to national circumstances by fine temporal and spatial scale and closer link between biomass and soil dynamics. However, this requires technical competence and scientific expertise (Palm, Ostwald, Berndes, & Ravindranath, 2009). Previous studies in general focused on the assessment of no‐project‐implemented carbon dynamics baselines based on empirical models or forest inventories (Dushku & Brown, 2003; Dutschke, Butzengeiger, & Michaelowa, 2006).

Selection of the model used in a project and the model parametrization and calibration are critical issues for proper documentation of the validity and completeness of the data. Diagnostic biosphere models using remote‐sensing data would be useful in CDM‐AR projects because of the complete coverage of information in the subjective study area, which enables the interpretation of land use and plant phenology. However, most conventional diagnostic biosphere models demonstrate low predictive skill and show uncertainty in simulations according to the quality of available satellite observations, and stress factors without biophysical processes (Sasai, Ichii, Yamaguchi, & Nemani, 2005). Prognostic biosphere models, or process‐based models, predict carbon dynamics in the biosphere based on biogeochemical processes in individual carbon components driven mainly by climate variability. Changes in carbon stocks in the five specified pools under various circumstances are predictable using process‐based models, but applicability of the models to CDM‐AR projects critically depends on appropriate model validation with ground observations.

Several studies have investigated validation schemes for prognostic biosphere models (e.g., Braswell, Sacks, Linder, & Schimel, 2005; Fox et al., 2009; Mo, Chen, Ju, & Black, 2008; Santaren, Peylin, Viovy, & Ciais, 2007; Trudinger et al., 2007), although these studies were designed to examine carbon exchange rates between the atmosphere and the biosphere, not carbon pools and growth in plant organs and soil. Cienciala and Tatarinov (2006) estimated aboveground woody biomass in managed forests using adjusted ecophysiological and nitrogen parameters. Zhao, Xiang, Peng, and Tian (2009) performed sensitivity analysis and prediction for each plant organ in a managed forest, comparing long‐term fir‐stand data. These studies were performed in managed forests where long‐term data reflect moderately stable conditions, which differ from the conditions under land use changes for recovering degraded ecosystems. Saito, Ito, and Maksyutov (2014) applied the assimilation scheme of a prognostic biosphere model to 10‐years average aboveground biomass and CO_2_ concentration data by combining it with an atmospheric tracer transport model, but they did not assess the temporal biomass changes or other carbon pools such as leaf and belowground biomass, the reporting of which is required in CDM‐AR projects. Unfortunately, there are few studies on model validation for carbon‐stock monitoring and growth processes based on field surveys.

In this study, we quantify the growth processes of carbon stocks in an afforestation project in China. This site has been implemented the “Grain for Green” program, which returns arable lands inappropriate for cultivation to forests, and restores degraded ecosystems to healthy condition. This program supplies increasing demands for timber in China and is expected to increase vegetation cover, leading to carbon sequestration (Liu, Li, Ouyang, Tam, & Chen, 2008). The adaptive monitoring of carbon pools following afforestation is necessary to assess the potential of plantations as carbon sinks. By monitoring the carbon pools in an afforestation project, we aim to estimate carbon fixation with adapting to ecophysiological changes and to address the uncertainty in predicting carbon‐stock changes and ecophysiological characteristics. We used an optimization scheme for biophysical parameters in a process‐based model with field surveys to represent plant growth following afforestation.

## 2 MATERIAL AND METHODS

### 2.1 Study site and biomass data

The study site is an 11‐year‐old *Eucommia ulmoides* plantation in Loess Plateau, consisting mostly of silt soil, in Lingbao City, Henan Province, China (34°16′N, 110°40′E, 1,000 m a.s.l). Annual average air temperature and annual precipitation for 1981–2010 measured at Lushi station, 40 km southeast of the site, are 13.7°C and 686 mm, respectively. *Eucommia ulmoides* is one of the common deciduous broadleaf trees in central and southern China. The trees at the study site were planted in 1999 on an abandoned cornfield as part of the Grain for Green program (Cao, Chen, & Yu, 2009; Wang, Hu, Deng, Shangguan, & Deng, 2018). This forest had been surveyed as a candidate site for a new afforestation project by a joint enterprise between China and Japan (Hitz, 2010).

To obtain biomass data, we conducted a field survey at the *E. ulmoides* plantation in 2009. We measured trunk diameter at breast height (*D*; m) and tree height (*H*; m) of *E. ulmoides* trees (all 11 years old). Survey of *D* and *H* in four quadrats in the plantation was performed at the same time of biometric 7 model‐tree survey. Sixty‐four samples from the four quadrats showed 10.5 ± 2.0 cm in *D* and 7.8 ± 1.8 m in tree height. By comparison of model and quadrat trees, averages of *D* and *H* of the model trees biased by 0.9 cm and 0.0 m from the averages of quadrat trees, respectively. These biases were enough small to decide these model trees as representatives in the plantation.

These seven model trees were harvested and separated into aboveground (trunks and branches) and belowground (coarse roots) woody parts, and leaves. The dry weight (kg) of each part was measured. We applied allometric relationships based on proportional relationships of biomass weight to *D* ^2^ and *D* ^2^ *H* (Niklas, 2004). In general, both the above‐ and belowground biomass have allometric relationships with *D* and *H* as follows:

(1) $$y=aD^{2}H+b$$

The relationship between leaf biomass and *D* is as follows:

(2) $$y=cD^{2}+d$$

The parameters *a*, *b*, *c*, and *d* for *E. ulmoides* in the present study were obtained using the measurements from the harvested trees fitted by linear regression using least squares method. The leaves of one of the trees were extremely damaged because of drainage water from a nearby building, so the leaf biomass data from that tree were not included, and the parameters for Equation (2) were obtained from the data of six trees.

To estimate above‐ and belowground and leaf biomass in the years prior to the field survey, we estimated *D* for previous years from the tree rings of the seven harvested trees. The tree trunks were cut at breast height, and we assumed that the tree‐ring diameter for each year was the same as *D* for that year. Measured average tree height of 1–11 year measured by trunk analysis was used for *H*. The above‐ and belowground and leaf biomass for each year were estimated from Equations (1) and (2) and the mean value for *D* of each year. The amounts of carbon in the aboveground (AC), belowground (BC), and leaf (LC) biomass were estimated by applying the measured percentage carbon content of 46% for above‐ and belowground biomass and 45% for leaves. The calculated mean carbon mass of cut trees (kg‐C) was converted into carbon density per unit land area (kg‐C/m^2^) using the total of *D* ^2^ and *D* ^2^ *H* acquired from measured *D* and *H* of all tree in four 10 × 15 m quadrats (supplied in data repository).

### 2.2 Model description

This study used the process‐based ecosystem model Biome‐BGC (Kimball, White, & Running, 1997; Running & Hunt, 1993; Thornton et al., 2002; Thornton & Rosenbloom, 2005; White, Thornton, Running, & Nemani, 2000) to simulate the biomass growth and carbon fixation of each plant organ. Biome‐BGC predicts the carbon fixation of leaf, stem, and root associated with the carbon allocation of photosynthetic products to each plant organ for six plant functional types: deciduous broadleaf trees, deciduous needle‐leaf trees, evergreen broadleaf trees, evergreen needle‐leaf trees, C3 grasses, and C4 grasses. The photosynthetic productivity in the model has been investigated and verified by various studies (e.g., Pietsch, Hasenauer, & Thornton, 2005; Ueyama et al., 2010; Wang, Bauerle, & Reynolds, 2008). Biome‐BGC allocates the photosynthetic production to each plant organ on the basis of carbon allocation parameters, and soil carbon is supplied through the litter from plant organs.

Biome‐BGC was driven with ecophysiological parameters, initial site information, and climate‐forcing data at a daily time‐step with point simulation at the site. The input parameters for ecophysiological characteristics for this study are shown in Table 1. The initial site information included the site characteristics of elevation (m), latitude (degree N), albedo, effective soil depth (m), first‐year maximum leaf and stem carbon (kg‐C/m^2^), atmospheric nitrogen deposition (kg‐N/m^2^), symbiotic and asymbiotic nitrogen fixation (kg‐N/m^2^), soil carbon content of pools (kg‐C/m^2^), soil nitrogen content of the mineral pool (kg‐N/m^2^), and soil texture. The nitrogen input rate from atmospheric deposition to the ecosystem at the site was fixed at 0.002 kg‐N m^−2^ y^−1^, which is the average value in broadleaf forests in various regions of China (Toda et al., 2010). Soil texture at the site was obtained from the literature (Li & Shao, 2006) as 5% sand, 68% silt, and 27% clay. The initial conditions for soil carbon and nitrogen density at the site were simulated by running the model over a 6,000‐year period with the default plant ecophysiological parameters of C4 annual grass and a nitrogen supply to the soil of 0.03 kg m^−2^ y^−1^ by fertilization, which was obtained from a survey of plantation managers. In this spin‐up simulation, 50% of the aboveground crop biomass was removed every year to represent harvest practice (Penman et al., 2003).

**Table 1:** Ecophysiological parameters used for simulations in this study

Ecophysiological parameters (Deciduous broadleaf forest; DBF)	Value	Source
Transfer growth period as fraction of growing season	0.2	Default for DBF in Biome‐BGC
Litter‐fall as fraction of growing season	0.2	Default for DBF in Biome‐BGC
Annual leaf and fine root turnover fraction (year^−1^)	1.0	Default for DBF in Biome‐BGC
Annual live wood turnover fraction (year^−1)^	0.7	Default for DBF in Biome‐BGC
Annual whole‐plant mortality fraction (year^−1^)	0.005	Default for DBF in Biome‐BGC
Annual fire mortality fraction (year^−1^)	0.0025	Default for DBF in Biome‐BGC
Allocation (new fine root C:new leaf C; ratio)	See Table 3	Optimized (default = 1.0)
Allocation (new stem C:new leaf C; ratio)	See Table 3	Optimized (default = 2.20)
Allocation (new live wood C:new total wood C; ratio)	0.10	Default for DBF in Biome‐BGC
Allocation (new root C:new stem C; ratio)	0.47	Measured (default = 0.23)
Current growth proportion	0.5	Default for DBF in Biome‐BGC
C:N of leaves (kg‐C/kg‐N)	26.0	Measured (default = 1.0)
C:N of leaf litter, after retranslocation (kg‐C/kg‐N)	49.0	Default for DBF in Biome‐BGC
C:N of fine roots (kg‐C/kg‐N)	42.0	Default for DBF in Biome‐BGC
C:N of live wood (kg‐C/kg‐N)	50.0	Default for DBF in Biome‐BGC
C:N of dead wood (kg‐C/kg‐N)	442.0	Default for DBF in Biome‐BGC
Leaf litter labile proportion	0.39	Default for DBF in Biome‐BGC
Leaf litter cellulose proportion	0.44	Default for DBF in Biome‐BGC
Leaf litter lignin proportion	0.17	Default for DBF in Biome‐BGC
Fine root labile proportion	0.30	Default for DBF in Biome‐BGC
Fine root cellulose proportion	0.45	Default for DBF in Biome‐BGC
Fine root lignin proportion	0.25	Default for DBF in Biome‐BGC
Dead wood cellulose proportion	0.76	Default for DBF in Biome‐BGC
Dead wood lignin proportion	0.24	Default for DBF in Biome‐BGC
Canopy water interception coefficient (LAI^−1^/d)	0.041	Default for DBF in Biome‐BGC
Canopy light extinction coefficient	0.7	Default for DBF in Biome‐BGC
All‐sided to projected leaf area ratio (ratio)	2.0	Default for DBF in Biome‐BGC
Canopy average specific leaf area (projected area basis)	See Table 3	Optimized (default = 30.0)
Ratio of shaded SLA:sunlit SLA (ratio)	2.0	Default for DBF in Biome‐BGC
Fraction of leaf N in Rubisco	0.08	Default for DBF in Biome‐BGC
Maximum stomatal conductance (projected area basis; m/s)	See Table 3	Optimized (default = 0.005)
Cuticular conductance (projected area basis; m/s)	0.00001	Default for DBF in Biome‐BGC
Boundary‐layer conductance (projected area basis; m/s)	0.01	Default for DBF in Biome‐BGC
Leaf water potential at start of conductance reduction (MPa)	−0.6	Default for DBF in Biome‐BGC
Leaf water potential at complete conductance reduction (MPa)	−2.3	Default for DBF in Biome‐BGC
Vapor pressure deficit at start of conductance reduction (Pa)	930.0	Default for DBF in Biome‐BGC
Vapor pressure deficit at complete conductance reduction (Pa)	4,100.0	Default for DBF in Biome‐BGC

Note

For sensitivity analysis (Section 2.42.4), we used the carbon allocation parameters for stem, leaf, and roots along with specific leaf area (SLA), maximum stomatal conductance (*g*_smax_), and vapor pressure deficits (VPDs), changing their values by ±30% and ±60% of the default.

Abbreviations: LAI, Leaf area index; SLA, specific leaf area.

**Figure 1:**
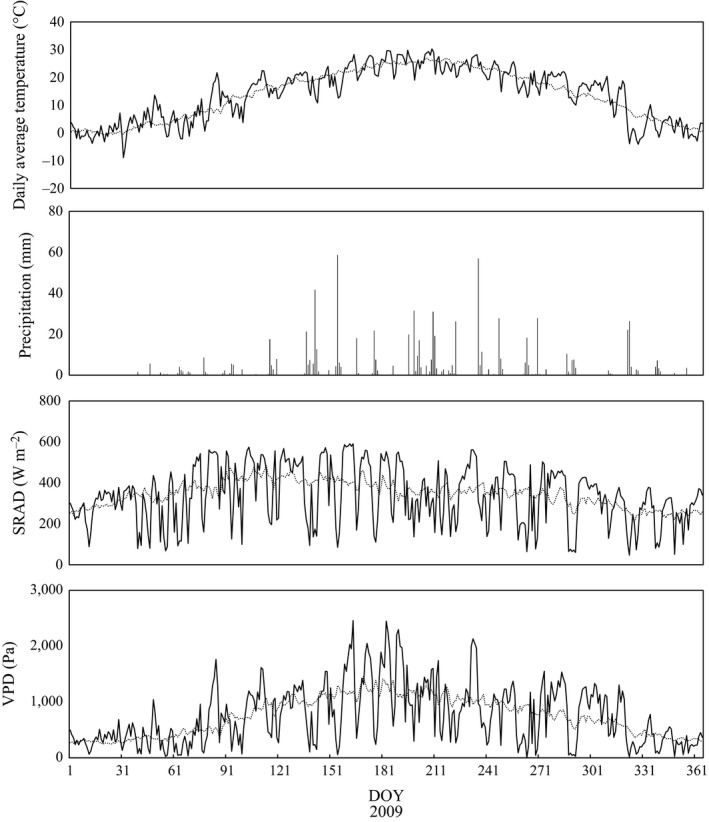
Meteorological data for daily average temperature (corrected by the mountain climate simulator MTCLIM), precipitation, solar radiation (SRAD), and vapor pressure deficit (VPD) generated from temperature, SRAD and VPD by MTCLIM for 2009 (solid lines). Dashed lines for SRAD and VPD represent the average values for 1981–2010

Biome‐BGC requires five climate‐forcing data; daily precipitation (cm), daily maximum and minimum air temperatures (°C), daylight average vapor pressure deficit (VPD; Pa), and daylight average shortwave radiant flux density (SRAD; W/m^2^). For this study, we used daily precipitation and maximum and minimum air temperatures at Lushi station for 1981–2010 from the National Climatic Data Center (NCDC), Climate Data Online (CDO) of the National Oceanic and Atmospheric Administration (NOAA) (NOAA, 2018, 2018). Daily VPD, SRAD, and day length were estimated from daily precipitation and maximum and minimum air temperatures using the mountain microclimate simulator MTCLIM 4.3 (Bohn et al., 2013; Kimball, Running, & Nemani, 1997; Running, Nemani, & Hungerford, 1987; Thornton, Hasenauer, & White, 2000; Thornton & Running, 1999) by taking into account the elevation and latitude at the site. The input daily average air temperature, precipitation, SRAD, and VPD calculated from observations at the nearest weather station Lushi in 2009 using MTCLIM are shown in Figure 1. The annual average air temperature, precipitation, SRAD, and VPD for 1981–2010 were 13.7°C, 692 mm, 346 W/m^2^, and 743 Pa, respectively. The peak daily mean temperature in 2009 was 30.3°C in July, and the lowest temperature was −8.9°C in January. The peak monthly precipitation was 156 mm in July. Vapor pressure deficit estimated from these meteorological values was highest in the summer. Estimated SRAD showed small variations around normal yearly values (210–484).

### 2.3 Sensitivity of carbon pool simulations to ecophysiological parameters

To represent the carbon storage and growth of each plant organ adaptively, parameter adjustment was required. We therefore analyzed the sensitivity of AC and LC to ecophysiological parameters related to photosynthesis and carbon allocation to plant organs to select the parameters for adjustment by an optimization scheme. The sensitivity of BC was not analyzed because BC is calculated by multiplying AC by the ratio of carbon allocation of coarse root carbon to stem carbon (CRC:SC) in Biome‐BGC, so BC can be reproduced by using the appropriate AC and the CRC:SC ratio.

In Biome‐BGC, the photosynthetic assimilation rate *A* (µmol CO_2_ m^−2^ s^−1^) is simulated using Farquhar's photosynthesis model (Farquhar, Caemmerer, & Berry, 1980; De Pury & Farquhar, 1997):

(3) $$A=\text{min}\left(A_{c},A_{j}\right),$$

where *A_c_* (μmol CO_2_ m^−2^ s^−1^) is the carboxylation‐limited assimilation and *A_j_* (μmol CO_2_ m^−2^ s^−1^) is the RuBP‐regeneration‐limited assimilation. *A_c_* and *A_j_* are calculated as follows:

(4) $$A_{c}=\begin{array}{c} \frac{V_{\text{cmax}}\left(C_{i}-\Gamma^{*}\right)}{C_{i}+K_{c}\left(1+O_{2}/K_{o}\right)}-R_{d} \end{array},$$

(5) $$A_{j}=\begin{array}{c} \frac{J\left(C_{i}-\Gamma^{*}\right)}{4.5C_{i}+10.5\Gamma^{*}}-R_{d}, \end{array}$$

where *V*_cmax_ (μmol CO_2_ m^−2^ s^−1^) is the maximum carboxylation rate, *J* (μmol CO_2_ m^−2^ s^−1^) is the actual electron transport rate and is related to the incident photosynthetic photon flux density (PPFD), and the maximum electron transport rate (*J*_max_; μmol CO_2_ m^−2^ s^−1^) (Kuehn & McFadden, 1969), *C_i_* (Pa) is the leaf intercellular CO_2_ concentration (partial pressure), Γ^*^ (Pa) is the CO_2_ compensation point in the absence of photorespiration, *K_c_* and *K_o_* are the Michaelis–Menten constants of Rubisco for CO_2_ and O_2_, respectively, and *R_d_* (μmol CO_2_ m^−2^ s^−1^) is the photorespiration rate.

Biome‐BGC calculates *V*_cmax_ from leaf nitrogen data and Rubisco activity as follows:

(6) $$V_{\text{cmax}}=\text{lnc}\times \text{flnr}\times \text{fnr}\times \text{act},$$

where lnc (kg‐N/m^2^) is the leaf nitrogen content per unit projected sunlight leaf area, flnr (kg‐N/kg‐N) is the proportion of leaf nitrogen content that is in Rubisco, fnr (kg‐Rubisco/kg‐N) is the weight ratio of Rubisco to its nitrogen content (=7.16), and act (μmol‐CO_2_ kg‐Rubisco^−1^ s^−1^) is the Rubisco activity, which is adjusted for temperature and O_2_ and CO_2_ levels.

*A* in Equation (3) can also be determined by using the relationship between the photosynthetic assimilation, the stomatal conductance, and the atmospheric and leaf intercellular CO_2_ concentrations as follows:

(7) $$A=g\left(C_{a}-C_{i}\right),$$

where *g* (μmol‐CO_2_ m^−2^ s^−1^ Pa^−1^) is the leaf‐scale conductance of CO_2_, and *C_a_* (Pa) is the atmospheric concentration of CO_2_. To represent the stomatal closure corresponding to environmental stresses, Biome‐BGC scales the maximum stomatal conductance (*g*_smax_) by a series of multipliers between 0 and 1 for stresses connected with the PPFD, soil‐water potential, minimum temperature, and VPD (Körner, 1995; White et al., 2000). Biome‐BGC represents the ecophysiological responses of stomata to the stresses of the light and water environment, and photosynthetic activity to nitrogen content, by these processes.

These processes are calculated for leaves both in sun and shade by daily steps. The actual photosynthesis can be calculated by solving the quadric expressions in Equations (3) and (7). In Biome‐BGC, 39 ecophysiological parameters are used to represent photosynthetic production based on *A* and its allocation to each plant organ according to specified allocation parameters. We conducted a literature search for these parameters (e.g., Chiesi et al., 2007; Cienciala & Tatarinov, 2006; Hidy et al., 2012; Jarvis, 1976; Jarvis & McNaughton, 1986; Leuning, 1995; Pietsch et al., 2005; Warren, Livingston, & Turpin, 2004; White et al., 2000). We then arbitrarily selected seven parameters for sensitivity analysis to determine the critical parameters for variability in the carbon pools of each plant organ. For carbon allocation parameters, we used the ratios of fine root carbon to leaf carbon (FRC:LC), stem carbon to leaf carbon (SC:LC), and CRC:SC. We also selected the specific leaf area (SLA), *g*_smax_, VPD at the final reduction of stomatal conductance (VPD*_f_*), and VPD at the initial reduction of stomatal conductance (VPD*_i_*). In the sensitivity analysis, we individually tested the sensitivity of predicted AC and LC to variations in parameter levels of ±30% and ±60% of default values.

### 2.4 Model tuning

**Figure 2:**
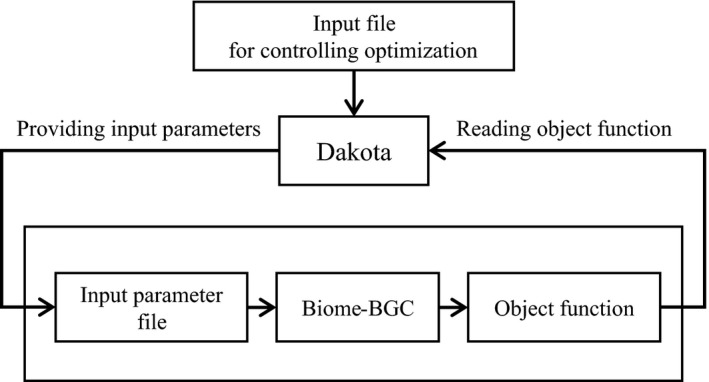
Schematic diagram of the system for minimizing the object function using Dakota optimizer (Adams et al., 2013). The Dakota system provides the parameters generated by user‐specified optimization methods to a model and then reads the object function estimated from model outputs. The system iterates this procedure until the object function is minimized

The seven parameters selected as described in Section 2.32.3 were optimized to represent the observed biomass growth using the Dakota optimizer version 5.3 (Adams et al., 2013). Dakota is an optimizer developed by Sandia National Laboratories and is applicable for optimizing model parameters using an interface in the Dakota system (Figure 2). Dakota can apply several optimization algorithms by simply rewriting the settings file. To optimize the ecophysiological parameters in this study, we applied the algorithms for derivative‐free global optimization methods for linear and nonlinear constraints in the Dakota system (Adams et al., 2013).

Five algorithms of derivative‐free global methods—the dividing rectangles (DIRECT) method of the common optimization library interface (coliny DIRECT), the DIRECT method of North Carolina State University library (NCSU DIRECT), the efficient global optimization method (EGO), the coliny evolutionary algorithm (coliny EA), and the Single‐Objective Genetic Algorithm (SOGA)—were tested to minimize the object function for reproducing carbon fixation of each plant organ. All optimization algorithms ran the model iteratively, revising the input parameters to minimize the object function and generate search‐optimized parameters. The derivative‐free global methods can search optimal parameters for the minimum object function even for black‐box models or functions without derivatives, and these five methods were able to solve nonconvex functions such as the Rosenbrock function (Adams et al., 2013). Trudinger et al. (2007) compared optimization methods for data assimilation of terrestrial ecosystem models, and they reported that the design of the object function is more important for parameter optimization than selection of optimization methods. In this study, we simply adopted the algorithm showing the highest performance of the five tested to represent the measured carbon density of plant organs. The results of the tests of the five algorithms using measured biomass data and minimized object functions (described in Equation 8 below) are shown in Table A1.

As the object function to be minimized by Dakota, we defined the average yearly relative error between simulated and measured LC and AC pools over 11 years as follows:

(8) $$f=\frac{1}{2\left(n-1\right)+W_{A}+W_{L}}\left\{\sum_{i=1}^{n-1}\sqrt{{\left(\frac{AC_{i,\text{sim}}-AC_{i,\text{obs}}}{AC_{i,\text{obs}}}\right)}^{2}}+\sum_{i=1}^{n-1}\sqrt{{\left(\frac{LC_{i}-LC_{i,\text{obs}}}{LC_{i,\text{obs}}}\right)}^{2}}+W_{A}\times \sqrt{{\left(\frac{AC_{n,\text{sim}}-AC_{n,\text{obs}}}{AC_{n,\text{obs}}}\right)}^{2}}+W_{L}\times \sqrt{{\left(\frac{\left(LC_{n,\text{sim}}-LC_{n,\text{obs}}\right)}{LC_{n,\text{obs}}}\right)}^{2}}\right\},$$

where *f* is the object function, *i* is stand age (years), *n* is the stand age at the last field survey (*n* = 11 in this study), AC*_i_* and LC*_i_* are the aboveground woody and leaf carbon densities in year *i*, respectively, and *W_A_* (=25) and *W_L_* (=5) are the weighting coefficients of the error in the last field survey year for aboveground woody and leaf carbon density, respectively. Subscripts sim and obs indicate simulated and observed values, respectively.

In addition to optimization using 11‐year data, we also performed optimization using 1‐ to 5‐year‐old stand data to investigate parameter variability during the afforestation and plant growth phases of the project. We performed optimization at three different phases with different parameters to represent carbon fixation during each phase: (i) the phase before implementation of the afforestation project (“planning” phase), using the default parameters; (ii) the first monitoring phase at year 5, with parameters optimized using stand data for years 1–5; and (iii) an updated monitoring phase at year 11, with parameters optimized using stand data for years 1–11. The field survey at year 11 was performed as part of this study and yielded the tree‐ring data and total *D^2^* and *D* ^2^ *H* for each quadrat; however, there was no field survey at year 5. Instead, we simply used the data for years 1–5 estimated from the allometric relationships and the tree‐ring measurements from the 11‐year‐old stand.

### 2.5 30‐year carbon fixation

In this study, we estimated the carbon fixation in each carbon pool over the project period (30 years as in some approved CDM‐AR monitoring methodology). The goal was to assess total carbon fixation and the variability associated with ecophysiological parameters that are individually tuned to three different phases as described in Section 2.42.4: before implementation, year 5 monitoring, and year 11 updated monitoring. We estimated the variability in carbon fixation of above‐ and belowground woody biomass and leaf biomass at the site for 30 years after afforestation using climate‐forcing data for 1981–2010. To reduce the impact of climate anomalies on plant carbon fixation in the simulation, the model was driven by repeating the 30‐year climate‐forcing data cyclically from the beginning of each year, with atmospheric CO_2_ concentrations for 1981–2010, and then averaging the resulting ensemble of estimated carbon pools. In addition to the simulation using the model fully tuned by the observations of eleven years, we estimated the carbon fixation using default parameters and those tuned with observations from the stand at ages 1–5 years. These processes yield simulations for the planning phase, with updated predictions for the implementation phase of an afforestation project.

## 3 RESULTS

### 3.1 Biomass variability and meteorological conditions at the study site

**Figure 3:**
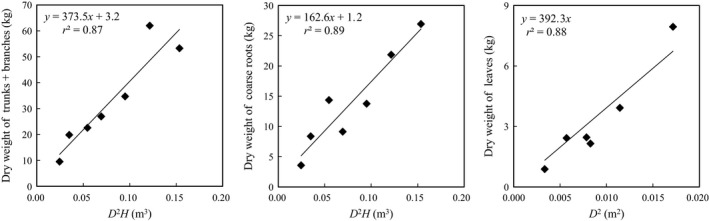
Allometric relationships between above‐ and belowground biomass and diameter at breast height for each tree trunk diameter (*D*) and tree height (*H*), and between leaf biomass and *D*, for 11‐year‐old *Eucommia ulmoides*

**Figure 4:**
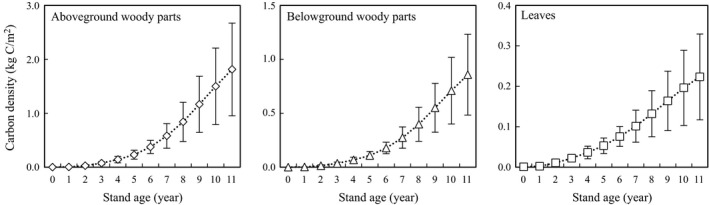
Yearly carbon biomass of each tree part per unit land area estimated from the field survey in year 11. Plotted values are averages from seven trees (for above‐ and belowground parts) or six trees (for leaves). Values for years 1–10 were calculated on the basis of tree‐ring analysis. The error bars show standard deviations as estimated from the variation of *D* in tree‐ring analysis

We determined the allometric relationships between *D* and *H* and the dry weights of aboveground woody biomass (trunks and branches), belowground woody biomass, and leaf dry weight for *E. ulmoides* using the data from the biometric survey (Figure 3). The relationships were well approximated by straight lines. The parameters *a* and *b* of Equation (1) for aboveground woody parts were 373.5 and 3.2, respectively; for belowground woody parts they were 162.6 and 1.2, respectively. The parameters *c* and *d* of Equation (2) for leaves were 392.3 and 0.0, respectively. The carbon densities (AC, BC, and LC) for years 1–11 were estimated using the total *D* ^2^ and *D* ^2^ *H* of the 11‐year‐old stand, the allometric relationships (Figure 3), the *D* ^2^ and *D* ^2^ *H* for each year as determined from ring analysis, and the measured fractional carbon content of aboveground woody parts (0.46) and leaves, (0.45; Figure 4). The carbon densities for AC, BC, and LC at year 11 were 1.81, 0.86, and 0.22 kg‐C/m^2^, respectively. These values were used as measured carbon pools for validation of the model. The standard deviations of estimated carbon densities were based on the variability of *D* in the tree‐ring analysis. The mean ratio of the belowground carbon density to the aboveground carbon density (trunks and branches) over 11 years was 0.47.

### 3.2 Sensitivity analysis of carbon pools to ecophysiological parameters

**Figure 5:**
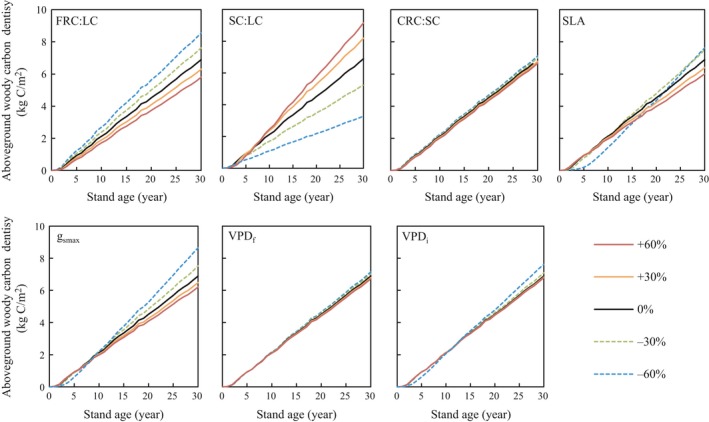
Sensitivity of aboveground woody‐carbon density to seven ecophysiological parameters. Curves represent the model predictions when parameters were changed by ±60%, ±30%, and 0% (default of Biome‐BGC). CRC, coarse root carbon; FRC, fine root carbon; *g*_smax_, maximum stomatal conductance; LC, leaf carbon; SC, stem carbon; SLA, specific leaf area; VPD*_f_*, vapor pressure deficit at the final reduction of stomatal conductance; VPD*_i_*, vapor pressure deficit at the initial reduction of stomatal conductance

**Figure 6:**
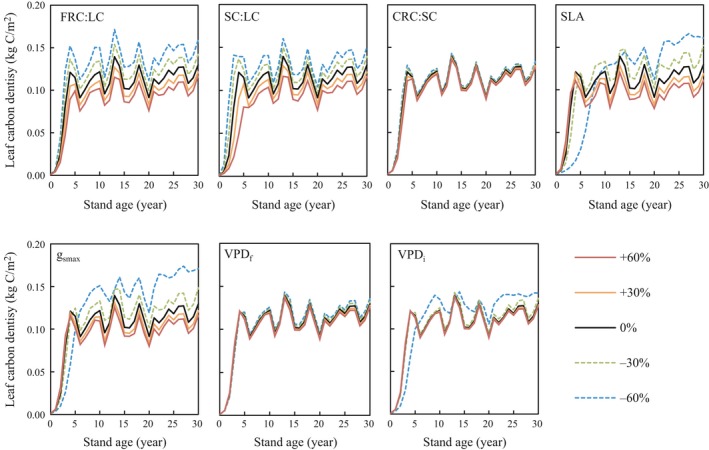
Sensitivity of leaf carbon density to seven ecophysiological parameters. Curves represent the model predictions when parameters were changed by ±60%, ±30%, and 0% (default of Biome‐BGC). See caption to Figure 5 for an explanation of the parameters

We investigated sensitivity of AC and LC to ecophysiological parameters by varying individual parameters by ±30% and ±60% from default values to select the parameters for optimization for reproducing measured biomass carbon (Figures 5 and 6). The estimates of AC and LC at the 30‐year stand age were 6.89 and 0.13 kg‐C/m^2^ using the default parameters. Both AC and LC at the 30‐year stand age varied with changes in the seven parameters, namely FRC:LC (5.80–8.56 and 0.12–0.16 kg‐C/m^2^, respectively), SC:LC (3.27–9.15 and 0.12–0.15 kg‐C/m^2^), CRC:SC (6.67–7.12 and 0.126–0.133 kg‐C/m^2^), SLA (6.01–7.62 and 0.12–0.17 kg‐C/m^2^), *g*_smax_ (6.20–8.65 and 0.11–0.16 kg‐C/m^2^), VPD*_f_* (6.71–7.15 and 0.13–0.14 kg‐C/m^2^), and VPD*_i_* (6.78–7.62 and 0.13–0.14 kg‐C/m^2^). These are the ranges of variation when the respective parameters were ±60% of default parameter values.

FRC:LC controls the allocation of photosynthetic production to fine root carbon and LC in the model, with LC increasing with decreasing FRC:LC. AC is estimated by multiplying LC by SC:LC in the model (Running & Hunt, 1993), so AC and LC show a high sensitivity to FRC:LC and SC:LC allocation parameters.

The sensitivity of the carbon pools to SLA reversed from the early to the later stages of the 11‐year plant growth period (note the line representing −60% of default SLA in Figures 5 and 6). Changes in *g*_smax_ strongly affected AC and LC, and the sensitivity of LC also reversed around year 5 at *g*_smax_ = −60% of the default value. Thus, the sensitivity of plant growth to ecophysiological parameters sometimes changes according to growth stages, and the update of model parameters using field survey data every few years is necessary for improving the adaptivity of the carbon fixation predictions.

Three parameters, CRC:SC, VPD*_f_*, and VPD*_i_*, had little effect on AC and LC. Although CRC:SC controls the carbon allocation ratio between coarse root and aboveground wood (stems or trunks), the range of estimated AC at 30‐year stand age was relatively narrow at 6.67–7.12 kg‐C/m^2^, even with changes of ±60% from default values. These results show that variability in the three parameters CRC:SC, VPD*_f_*, and VPD*_i_* had negligible effects on carbon pool estimates in our studies, and thus, CRC:SC was set to the measured ratio of BC to AC averaged over eleven years (0.47), and we used the default parameter values for deciduous broadleaf forest for both VPD*_f_*, and VPD*_i_*. In this study, therefore, we selected four parameters, FRC:LC, SC:LC, SLA, and *g*_smax_, to fit estimates of AC and LC to the observations.

### 3.3 Model tuning

We tested derivative‐free global methods in Dakota to adjust the selected parameters to represent the measured biomass growth of each plant organ. The value of the object function minimized by coliny DIRECT, which showed the best performance, was 0.118. Table 2 shows a comparison between the results using nonweighted and weighted object functions and AC and LC at 11 years. The minimized nonweighted (*W_A_* = *W_L_* = 1) object function in Equation (8) using 1‐ to 11‐year‐old stand data was 0.225, whereas the weighted (*W_A_* = 25, *W_L_* = 5) object function was 0.118, where the best weight coefficients were determined by trial and error. The weight factors were not calibrated using the optimization method because of the computational cost. The weight functions we used are designed to prioritize the information from the latest field survey. The model simulation optimized with the weighted object function showed a closer match to the observed AC and LC, showing that updates using the latest field survey data can improve the carbon pool estimates at an afforestation site.

**Table 2:** Comparison of observed and simulated aboveground woody and leaf carbon density for the year of the field survey (year 11) using the optimized models with nonweighted and weighted object functions using the coliny DIRECT method of Dakota (Adams et al., 2013)

	Carbon density (kg‐C/m^2^)		Minimized object function
	Aboveground woody (relative error)	Leaf (relative error)	
Observed	1.812	0.223	
Simulated with nonweighted object function	1.815 (0.002)	0.173 (0.226)	0.225
Simulated with weighted object function	1.812 (0.000)	0.174 (0.220)	0.118

The four selected ecophysiological parameters were adjusted to fit the observed biomass variability for the first 5 years and for the entire 11‐year period (Table 3). All parameters showed variations relative to the default values as a result of tuning the model. Three of the adjusted parameters showed differences between the first 5 years and entire 11‐year period: FRC:LC, SC:LC, and *g*_smax_. The adjusted FRC:LC and *g*_smax_ for the first 5 years (2.36 and 0.008 m/s, respectively) increased in magnitude relative to the default settings (1.0 and 0.005 m/s), whereas those for the entire 11 years (0.53 and 0.003 m/s) were lower. The adjusted SC:LC was lower that the default (2.20) for both the first five years (1.23) and the entire 11 years (1.76). These results show that the appropriate values for ecophysiological parameters can vary with stand age. Specific leaf area adjusted for both the 5‐year and 11‐year periods (15 m^2^/kg‐C) was half the default value (30 m^2^/kg‐C).

**Table 3:** Default ecophysiological‐characteristic parameters and those tuned by the coliny DIRECT method of Dakota (Adams et al., 2013) using the biomass data in the first 5 years and the entire 11‐year period

	Default	5‐year	11‐year
FRC:LC (ratio)	1.00	2.36	0.53
SC:LC (ratio)	2.20	1.23	1.76
SLA (m^2^/kg‐C)	30.00	15.00	15.00
*g*_smax_ (m/s)	0.005	0.008	0.003

Abbreviations: FRC, fine root carbon; *g*_smax_, maximum stomatal conductance; LC, leaf carbon; SLA, specific leaf area.

**Figure 7:**
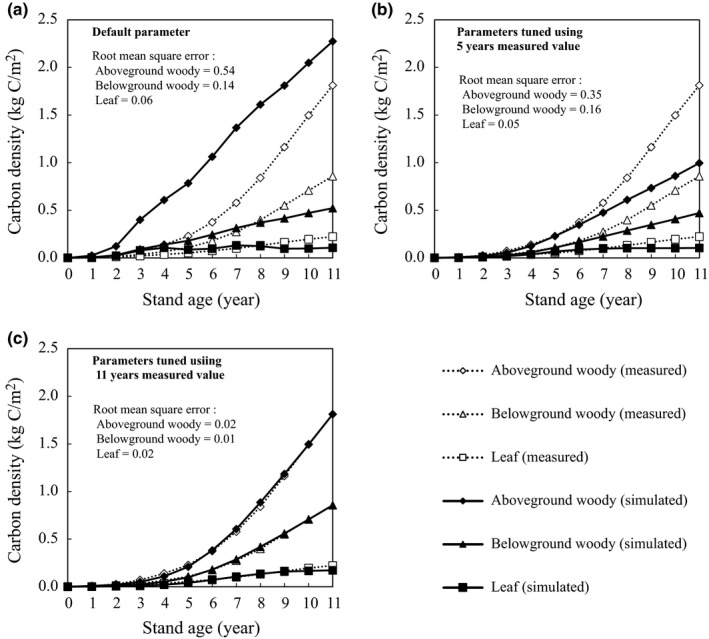
Measured above‐ and belowground woody biomass carbon and leaf biomass carbon, and those simulated using (a) the default ecophysiological‐characteristic parameters, (b) parameters tuned by Dakota using the observed biomass in the first 5 years, and (c) parameters tuned using the entire 11‐year period

When using the default settings, AC was overestimated for the entire 11‐year period and reached 2.27 kg‐C/m^2^ at the stand age of 11 years (Figure 7a). The root mean square error (RMSE) between the observed and estimated AC was 0.54 kg‐C/m^2^. The estimates using default settings overestimated LC and BC by the stand age of 7 years and then switched to an underestimate after 9‐year stand age, with the discrepancies between the observations and the estimates expanding every year. The RMSEs for LC and BC between observations and estimates with default settings were 0.06 and 0.14 kg‐C/m^2^, respectively. The estimates using parameters adjusted for the first 5 years fitted the observed AC, LC, and BC by 5‐year stand age, but underestimated them after 6‐year stand age (Figure 7b). RMSEs for AC, LC, and BC for years 1–5 were 0.01, 0.01, and 0.01 kg‐C/m^2^, respectively; for years 6–11 they were 0.35, 0.05, and 0.16 kg‐C/m^2^, respectively; and for the entire 11 years they were 0.35, 0.05, and 0.16 kg‐C/m^2^, respectively. AC, LC, and BC estimated using the parameters adjusted for the entire 11‐year period agreed well with observations (RMSE = 0.02, 0.02, and 0.01 kg‐C/m^2^, respectively; Figure 7c).

### 3.4 30‐year carbon fixation

**Figure 8:**
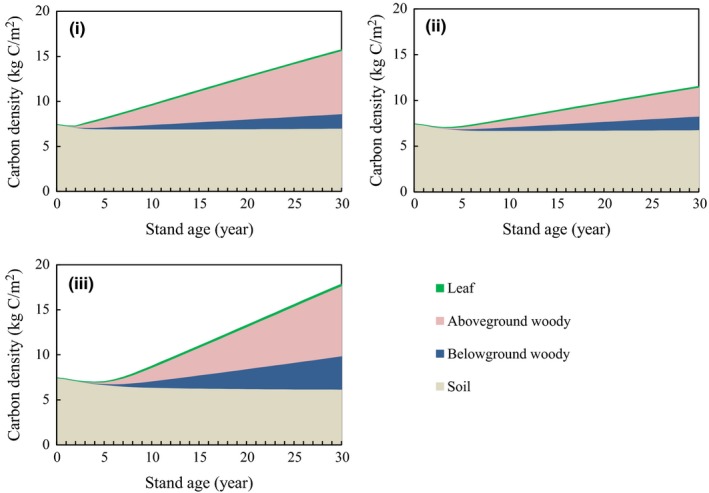
Temporal changes in leaf, above‐ and belowground woody, and soil carbon density simulated using (a) the default ecophysiological‐characteristic parameters, (b) those tuned using data from the first 5 years, and (c) those tuned using data from the entire 11‐year period of the implementation phase of the plantation project (30 years). The values are the ensemble averages of simulation runs with cyclic use of 30‐year meteorological data

We estimated the carbon fixation for 30 years after planting at the afforestation site to assess the impact on carbon pool estimates of using ecophysiological parameters that were individually optimized for three different project phases (see Section 2.52.5; Figure 8 and Table 4). We also estimated the carbon fixation in the soil pool. The soil carbon pool is affected by estimates of AC, BC, and LC because the soil pool varies with the carbon supplied through litter. There were large differences in plant biomass and total carbon pools estimated with the three parameter settings, namely the default values (phase i), those adjusted for the initial 5 years (phase ii), and those adjusted for the entire 11 years (phase iii). The average fixation rates of leaf and soil carbon (0.004–0.006 and −0.04 to −0.01 kg‐C m^−2^ year^−1^, respectively) were very low compared to AC (0.11–0.26 kg‐C m^−2^ year^−1^). The total carbon density at the end of the 30‐year project estimated by the model tuned during the first 5‐year period (11.55 kg‐C/m^2^) was approximately 30% lower than that from the model tuned for the 11‐year period (17.86 kg‐C/m^2^); this result shows the variability of carbon fixation rates and ecophysiological parameters between project phases. The total combined carbon fixation of AC, BC, LC, and soil as estimated using parameters optimized for each phase ranged from 4.2 to 10.5 kg‐C/m^2^ in a 30‐year projection (Figure 8).

**Table 4:** Carbon density in the forest carbon pools at the end of the 30‐year afforestation project, and average carbon fixation rates over the 30 years as estimated (i) using the default ecophysiological‐characteristic parameters (as in the planning phase), (ii) using parameters tuned with data for the first 5 years after planting, and (iii) using parameters tuned using data from the entire 11‐year period of the implementation phase

	Average ± standard deviation of carbon density at the end of 30‐year project (kg‐C/m^2^)			Average carbon fixation rates for 30 years (kg‐C m^−2^ y^−1^)		
	(i)	(ii)	(iii)	(i)	(ii)	(iii)
Leaf	0.12 ± 0.01	0.10 ± 0.01	0.19 ± 0.01	0.004	0.003	0.006
Aboveground woody	7.00 ± 0.08	3.17 ± 0.06	7.80 ± 0.07	0.23	0.11	0.26
Belowground woody	1.61 ± 0.02	1.50 ± 0.03	3.69 ± 0.03	0.05	0.05	0.12
Soil	7.02 ± 0.02	6.78 ± 0.02	6.18 ± 0.05	−0.01	−0.02	−0.04
Total	15.75 ± 0.11	11.55 ± 0.08	17.86 ± 0.08	0.28	0.14	0.35

Note

The carbon density values at 30 years are the ensemble averages of simulation runs with the cyclic use of 30‐year meteorological data beginning in each year. Standard deviations are also shown.

## 4 DISCUSSION

### 4.1 Parameter variation with plant growth stage

The ecophysiological parameters FRC:LC, SC:LC, SLA, and *g*_smax_, having shown a high contribution to AC and LC, were optimized to reproduce the temporal changes in carbon pools for each plant organ. They also showed variability with plant growth stage. FRC:LC optimized using the data from years 1 to 5 was higher (2.36) than the default (1.0), and that optimized by 1–11‐year data was lower (0.53). This difference reflects a change in growth patterns where the production of fine roots, and increases in root length, are greater in younger forests (Grier, Vogt, Keyes, & Edmonds, 1981). The mean and standard deviation of FRC:LC in the data collected for general deciduous broadleaf forest of Biome‐BGC were both 1.2 (White et al., 2000), and FRC:LC at phases ii and iii in our study fell within this range. Ellenberg, Mayer, and Schauermann (1986) reported similar values (0.55) in *Fagus* stands.

Values of SC:LC optimized using 1‐ to 5‐year and 1‐ to 11‐year data were both lower (1.23, 1.76, respectively) than the default value (2.20). These lower values mean that the proportional allocation of carbon to leaf compared to stem was higher than that for a generalized deciduous broadleaf forest in Biome‐BGC. This observation might reflect the phenomenon where carbon allocation to leaf becomes higher in arid conditions (Orians & Solbrig, 1977). The mean SC:LC value and standard deviation of the collected data for Biome‐BGC were 2.20 and 1.10, respectively (White et al., 2000). The optimized values in this study fell within this range. Several other studies reported similar values in deciduous broadleaf stands (e.g., *Betula pubescens* [White et al., 2000], *Fagus crenata* [Kakubari, 1977; White et al., 2000]).

BC was reproduced by using the ratio of BC to AC as CRC:SC (0.47) estimated from field survey data, which is higher than the default value (0.23). This demonstrated that the coarse root growth corresponded to a higher allocation of photosynthetic production in roots with the response of trees to water stress (Silva et al., 2004). CRC:SC had a small contribution to AC and LC, but this parameter is primarily important for reproducing BC. Because CRC:SC did not affect AC or LC, we were able to simply use the ratio of measured AC and BC. If CRC:SC had affected AC and LC by supplying carbon and nitrogen through litter, then CRC:SC should have been considered as a parameter to optimize.

Specific leaf area as optimized using 5‐year and 11‐year data showed a lower value (15 m^2^/kg‐C) than the default value for deciduous broadleaf forests (30 m^2^/kg‐C). In semi‐arid and arid areas, leaves protect themselves from water stress and reduce transpiration by thickening (Orians & Solbrig, 1977). *Eucommia ulmoides* is known to accumulate trans‐polyisoprene gum in its tissues (Nakazawa et al., 2009) and that could have influenced leaf thickness. The mean SLA and standard deviation from the collected data for Biome‐BGC were 32 and 11 m^2^/kg‐C, respectively (White et al., 2000). The optimized SLA in this study was somewhat lower than this range. This can be explained by the common tendency of leaf thickening in arid climates (Onoda et al., 2011). Specific leaf areas in deciduous broadleaf stands have been reported as 16.8 m^2^/kg‐C in an *Ulmus americana* stand (Reich, Kloeppel, Ellsworth, & Walters, 1995) and 16.3 m^2^/kg‐C in a *Godmania macrocarpa* stand (Holbrook, Whitbeck, & Mooney, 1995).

The *g*_smax_ optimized using 5‐year data was higher (0.008) than the default value (0.005) but lower than the default using 11‐year data (0.003). There are few data for *g*_smax_, and just one reference (Kelliher, Leuning, Raupach, & Schulze, 1995) was used in Biome‐BGC. Plants under semi‐arid and arid conditions in general survive by reducing transpiration through lower stomatal conductance (Orians & Solbrig, 1977). In the 11‐year‐old stand, transpiration increased with increased LC and leaf area, so leaves may have reduced transpiration by decreasing *g*_smax_.

These results show that our optimized parameters fell within actual ranges reported by other studies, and the values at each phase reflect the behavior of plants in semi‐arid and arid conditions. In this study, the parameters for reproducing the phenomena under semi‐arid conditions were selected a priori for the sensitivity analysis. That selection contributed to the reproducibility of the carbon pools, but at the same time it might have been arbitrary.

### 4.2 Monitoring method for afforestation projects in this study

We demonstrated a monitoring methodology for afforestation projects using process‐based model and field surveys. Conventional monitoring (e.g., AR‐ACM0003 of CDM [Methodology for Afforestation and Reforestation of Lands except Wetlands]; UNFCCC, 2013) includes field surveys every few years for monitoring plant carbon pools and enhanced QA/QC. This study used the mandatory field survey for model tuning, and the monitoring method that we propose is additionally applicable to conventional methods.

There are few studies using a process‐based model in the forest management field because of the requirement for large amounts of information that are not readily available or easily obtained (Landsberg, Johnsen, Albaugh, Allen, & McKeand, 2001). It is therefore beneficial to have model parameter information to reproduce plant growth. The model, once tuned, would be reusable for the next project or another project with the same tree species and climate conditions. Adaptive monitoring by using a tuned model with field surveys could help obtain sustainable ecosystem services.

The model parameter optimization scheme demonstrated that it is possible to adaptively estimate each plant carbon pool in 1‐ to 11‐year‐old stands (Figure 7c). The parameters describing ecophysiological characteristics changed with the three phases of the project, and there was uncertainty in the prediction of carbon fixation for 30 years using the parameters optimized for each phase (Figure 8 and Table 4). A reliable monitoring method that incorporates the uncertainties at each plant growth stage promotes positive outcomes of afforestation projects, such as acquiring carbon credits through market mechanisms. Reducing the uncertainty of the prediction using ecophysiological parameters requires the validation of ecophysiological characteristics by field survey every few years. Because this method requires a field survey every few years, it is consistent with the actual implementation of projects. Increasing the monitoring frequency can greatly contribute to the reduction of uncertainty in young stands if projects can afford the monitoring costs.

The process‐based approach can support forest inventory‐ and literature‐based approaches adapting changes of growth condition, noncommon species, and observation gap. Zhou et al. (2014) demonstrated the relationship between the biomass carbon density and forest age on a plantation by using the forest inventory and estimated carbon storage well. However, they also mentioned some uncertainties. For example, using a relationship established at a provincial scale and the data from the national forest inventory for only one phase of forest growth can easily cause error. The other uncertainties arose because the calculation of carbon storage resulted from the hypothesis that plantation growth followed the growth equation, and some plantings were affected by geographic and climate conditions. Models incorporating climate as a driving force and ecophysiological processes can mitigate these problems because they accommodate environmental changes.

As the next step resulting from this study, the process‐based approach could be used in conjunction with forest management models in assessing both CO_2_ mitigation and other ecosystem services concerned with forest management strategy and human activity. Forest management models can simulate carbon storage, plant growth, timber volume, and management effects of thinning and cutting (Lemma, Kleja, Olsson, & Nilsson, 2007; Ooba, Hayashi, Machimura, & Matsui, 2014). The growth process in these models is estimated on the basis of yield tables and forest inventory without accounting for changes in climate and environmental conditions. We focused on required monitoring, and thus, we estimated carbon fixation and uncertainty by using a process‐based model that calculated photosynthetic productivity based on meteorological forcing data and ecophysiological parameters. This can address concerns about the effects of changes in climate and environmental conditions, and it can also incorporate limited accessible data such as that for *E. ulmoides* stands planted under changing land use. The monitoring method used in this study can contribute to afforestation projects such as CDM‐AR and other market mechanisms that strictly require highly reliable monitoring.

### 4.3 Limitations of this study

Most studies assessing carbon storage in CDM‐AR projects have generally focused on no‐project‐implemented baseline carbon storage (e.g., Dushku & Brown, 2003), and there are no studies assessing CDM‐AR with long‐term monitoring using a process‐based model. Our approach requires regular field surveys to ensure the validation of the model, resulting in increases in the total cost of the project. This is a bottleneck in our approach; it would be acceptable, however, in situations where the afforestation project is aimed at carbon fixation and field surveys are obligatory.

The object function using relative error and weighting coefficients for AC and LC was designed to reproduce each plant organ pool (Equation 8). The weighted AC and LC for the year of the field survey were applied, and their values were determined by trial and error. We prioritized LC at year 11 (the year of the field survey) by a factor of 5 over the other years, and AC at year 11 by a factor of 25, on the basis of the ratio of their absolute values (in Equation 8), but these weight factors could vary at other sites.

In this study, the available data were restricted to trunk diameter at breast height, tree height, and the dry weight of each carbon pool in an 11‐year‐old *E. ulmoides* stand. We estimated AC, BC, and LC using the allometric relationships based on these data, but the period for which biomass data were available was shorter than the project term (30 years in CDM‐AR). This data limitation might also affect the spatial representativeness of our study. We focused mainly on our ability to reproduce carbon storage and changes in each plant organ during the afforestation monitoring phases by tuning the model, although the period with available data was insufficient to make this assessment over the project term. The growth rates and the ecophysiological conditions might change for years 12–30. Our methods were designed to estimate carbon fixation in the plantation after a land use change and to investigate the uncertainty in the model predictions by model tuning at each plant growth stage, but more observations over a longer period are needed to support our results.

Our method adaptively updates the prediction by observations every few years, which corresponds to long‐term predictions under limited conditions that do not cause phenomena beyond the model representation (fire and wind disturbances, exceptional drought, forest fire, pest attack). Biome‐BGC has no capacities responding to drastic environmental changes. For sustainable management and monitoring of afforestation, the incorporation of processes for above phenomena or the coupling with other management models is required.

## 5 CONCLUSIONS

To meet the requirement of highly reliable monitoring of afforestation projects aimed at mitigating CO_2_ levels, we have described a monitoring methodology that combines a process‐based ecosystem model simulation with field surveys for monitoring different phases of a project. The requirements of the model for afforestation projects were that the model reproduces carbon storage and changes in each plant organ using adjustable parameters, as well as defining the uncertainty of prediction using adjusted parameters and environmental conditions.

We demonstrated that the model can reproduce carbon density for AC, BC, and LC using optimized ecophysiological model parameters (FRC:LC, SC:LC, SLA, and *g*_smax_) for Biome‐BGC as a case study in a *E. ulmoides* plantation in Lingbao City, Henan Province, China. The ecophysiological parameters were optimized by a deviation‐free global optimization method, and the values of the optimized parameters changed with plant growth stages. The changes in parameters were consistent with the general behavior of plants under semi‐arid and arid climate conditions. The predicted carbon fixation for a 30‐year afforestation project using parameters optimized at each phase showed uncertainty. The total combined carbon fixation of AC, BC, LC, and soil estimated by parameters optimized for each phase was in the range of 4.2–10.5 kg‐C/m^2^ for the 30‐year project. Updating the parameters using field surveys every few years is important for reducing the uncertainty of the estimates and for determining the changes in ecophysiological characteristics at each plant growth stage.

This study shows how to estimate each plant carbon pool and understand the parameter changes with plant growth stages and the uncertainty of predicted carbon fixation in a plantation by an optimization scheme using field survey data. We expect the application of this monitoring method to support the management of afforestation projects by carbon fixation estimation adapting to observation gap, noncommon species, and variable growing conditions such as climate change, land use change.

## CONFLICT OF INTEREST

None declared.

## AUTHOR CONTRIBUTIONS

T. Miyauchi and T. Machimura conceived the ideas and designed the study; T. Miyauchi performed model simulation and analysis, and implementation of optimization scheme; T. Miyauchi wrote the manuscript; M. Saito corrected the manuscript.

## Data Availability

The biometric data for allometric relationships of *E. ulmoides* and the files related to optimization and simulation results are available on Zenodo (https://doi.org/10.5281/zenodo.2815612). The meteorological data for model input were download from NCDC CDO (https://www7.ncdc.noaa.gov/CDO/cdoselect.cmd?datasetabbv=GSOD&countryabbv&georegionabbv).

## APPENDIX 1

**Table A1:** Optimized parameters and the minimized object function determined by five algorithms for derivative‐free global optimization methods: the dividing rectangles (DIRECT) method of the common optimization library interface (coliny DIRECT), the DIRECT method of North Carolina State University library (NCSU DIRECT), the efficient global optimization method (EGO), the coliny evolutionary algorithm (coliny EA), and the single‐objective genetic algorithm (SOGA)

	Coliny DIRECT	NCSU DIRECT	EGO	Coliny EA	SOGA
FRC:LC	0.53	1.26	1.58	0.56	0.58
SC:LC	1.76	1.91	2.28	1.25	1.59
SLA	15.0	15.0	20.3	10.5	8.8
*g*_smax_	0.003	0.005	0.005	0.003	0.007
Object function	0.118	0.165	0.190	0.166	0.123

Abbreviations: FRC, fine root carbon; *g*_smax_, maximum stomatal conductance; LC, leaf carbon; SC, stem carbon.
